# IL-1β promotes A7r5 and HASMC migration and invasion via the p38-MAPK/Angpt-2 pathway

**DOI:** 10.1186/s40001-022-00781-1

**Published:** 2022-08-17

**Authors:** Anyu Xu, Jingchun Pei, Yunhong Yang, Baotong Hua, Jing Wang

**Affiliations:** 1 Department of Geriatric Cardiology, First Affiliated Hospital of Kunming Medical University, Kunming, 650500 Yunnan China; 2 Department of Neurosurgery, First Affiliated Hospital of Kunming Medical University, Kunming, 650500 Yunnan China

## Abstract

The migration, proliferation, and inflammatory factor secretion of vascular smooth muscle cells (VSMCs) are involved in the important pathological processes of several vascular occlusive diseases, including coronary atherosclerosis (CAS). Interleukin 1β(IL-1β), as a bioactive mediator of VSMC synthesis and secretion, can promote the pathological progress of CAS. In this study, we further explored the underlying molecular mechanisms by which IL-1β regulates VSMC migration, invasion. We pretreated A7r5 and HASMC with IL-1β for 24 h, and measured the expression of IL-1β, proliferating cell nuclear antigen (PCNA), cyclin D1, matrix metalloproteinase 2 (MMP2) and matrix metalloproteinase 2 (MMP9) in the cells by Western blotting. Cell migration and invasion ability were measured by Transwell and wound healing assays. Cell viability was measured by an MTT assay. We found that IL-1β upregulated the expression of proliferation-related proteins (PCNA and Cyclin D1) in A7r5 and HASMC, and induces the secretion of MMP2 and MMP9, promotes cell invasion and migration. In addition, in A7r5 and HASMCs treated with IL-1β, the expression of Angiopoietin-2 (Angpt-2) increased in a time-dependent manner, transfection with si-Angpt-2 suppressed cell migration and invasion, with downregulated MMP2 and MMP9 expression. Parallelly, we further found that the p38-MAPK pathway is activated in cells induced by IL-1β, p38-MAPK inhibitors can down-regulate the expression of Angpt-2. Collectively, these data demonstrated that IL-1β promotes A7r5 and HASMC migration and invasion via the p38-MAPK/Angpt-2 pathway.

## Introduction

CAS is a complex and perpetuating metabolic disease caused by the interaction of genetic and environmental risk factors, which is the main cause of various cardiovascular diseases including ischemic heart disease, ischemic stroke and coronary heart disease [1, 2]. The whole process of CAS involves a variety of cell morphology and function changes [3, 4]. Among these, the proliferation and migration of vascular smooth muscle cells (vascular smooth muscle cells, VSMCs) as a critical factor in the pathogenesis of CAS [5, 6]. Therefore, further exploration of the mechanisms regulating the proliferation and migration of VSMCs is of great significance to the prevention and treatment of CAS.

The accumulation of lipids and inflammatory cells in the blood vessel wall is the main feature of CAS. Inflammatory cells accumulate in the arterial wall and promote the proliferation and migration of VSMC cells by secreting inflammatory factors and expressing proteins [7–9]. Previous studies confirmed that the pro-inflammatory properties of IL-1β are related to the development of CAS lesions. As a growth factor for the proliferation of VSMCs, IL-1 can be paracrine and autocrine in VSMCs, leading to VSMCs proliferation and inflammation [10]. In parallel, it has been reported that IL-1β promotes the proliferation and inflammation of VSMCs by up-regulating the P2Y2 receptor (P2Y2R), and accelerates the process of atherosclerosis [11]. Nonetheless, the molecular mechanisms by which IL-1β promotes VSMCs proliferation and enhances CAS progression are poorly understood.

The signal cascade activated by mitogen-activated protein kinase (MAPK) can regulate various cell activities such as cell proliferation and migration by responding to extracellular stimuli [12]. p38-MAPK is essential for mediating IL-1β-induced inflammatory stress [13]. It was reported that the characteristic of IL-1β to initiate inflammatory stress is due to the activation of the p38/MAPK signaling pathway [12]. In addition, through proteomic analysis, it was found that IL-1β played a similar role to VEGF in human umbilical vein endothelial cells by activating the MAPKs signaling pathway [14]. Thus, IL-1β promotes the proliferation and migration of VMSCs may be related to the activation of p38-MAPK. In this study, an in vitro models of rat thoracic aortic smooth muscle cells (A7r5) and human aortic vascular smooth muscle cells (HASMCs) were used to study the involvement of the p38-MAPK pathway in the promotion of cell proliferation and migration by IL-1β, and its possible molecular mechanism.

## Materials and methods

### Reagents and antibodies

IL-1β was purchased from R&D systems (Minneapolis, MN). Antibodies IL-1β, cyclin D1, PCDNA, Angpt-2, MMP2, MMP9 were purchased from Santa Cruz Biotechnology (CA, USA). Antibodies p-p38, p38 were purchased from Abcam (MA, USA). MTT assay kit bought from Keygen Biotech (Nanjing, China). crystal violet staining solution was purchased from Sangon Biotech (Shanghai, China). The p38-MAPK inhibitor SB203580 was purchased from Cell Signaling Technology (Danvers, MA).

### A7r5 and HASMCs cells culture

According to the method of Wu et al. [15]. Briefly, A7r5 VSMCs and HASMc VSMCs were bought from the Shanghai Cell Bank of the Chinese Academy of Sciences (Shanghai, China). A7r5 and HASMc cells were cultured in DMEM medium containing 10% fetal bovine serum (Hyclone Co., Logan, UT, USA) with 1% streptomycin–penicillin (Thermo Fisher Scientific, Inc., USA).

### A7r5 and HASMCs cells viability

A7r5 and HASMCs cells viability was determined using the MTT Cell Proliferation and Cytotoxicity Assay Kit (MTT) in brief, cells were cultured with 10 ng/mL IL-1β for 24 h. After that, MTT reagent was added to each well and cells were incubated for another 4 h following the manufacturer’s instructions. Following incubation, the MTT solution was removed and 200 µl of DMSO solution was added to cells. Finally, the absorbance of each wells at 490 nm was measured using a microplate reader.

### Transwell assay

Transwell chamber with 8.0 μm pores (Corning, USA) was used to detect A7r5 and HASMCs cells migration ability. Briefly, seeded different treatments of A7r5 and HASMC into the upper chamber of the Transwell chamber, serum-free medium was added to lower chambers. After incubation for 12 h, the cells remaining on the upper surface of the membrane were removed with cotton swabs. The cells in the lower chamber were fixed with 4% paraformaldehyde and stained with crystal violet staining for 15 min. Images were obtained using an inverted fluorescence microscope (magnification × 100), and the cells of through the Transwell were counted using the ImageJ software.

### Wound healing assay

Wound healing assays were applied to detect cellular abilities of migration. Briefly, seeded different treatments of A7r5 and HASMC into 6-well plate, the monolayer was scratched with a sterile 10-μL pipette tip. Wound closure was observed after 0 and 24 h. Images were obtained using a microscope (magnification × 400), and analyzed with ImageJ software.

### Western blotting assay

Protein extracts from A7r5 and HASMC cells were prepared in RIPA buffer. The extracted protein samples (15 μg) were separated on 10% SDS-PAGE gels and transferred to polyvinylidene difluoride membranes. After blocking with 5% skim milk, the membranes were incubated with primary antibodies (IL-1β, cyclin D1, PCDNA, Angpt-2, MMP2, MMP9, p-p38, p38) followed by secondary antibodies. Immunoblots were developed using the chemiluminescence system (ECL kit, Amersham) according to the manufacturer’s instructions, analyzed with Image J software (NIH, Bethesda, MD, USA).

### Statistical analysis

All data were analyzed using the t test, and presented as mean ± SD. Statistical analyses were performed using Prism GraphPad Software (GraphPad Prism 7, GraphPad Software Inc). *P*-value < 0.05 (^*^, ^#^) or P-value < 0.01(^**^, ^##^) was considered statistically significant.

## Results

### *IL-1β treatment promotes the proliferation of A7r5 and HASMCs *in vitro

**Fig. 1:**
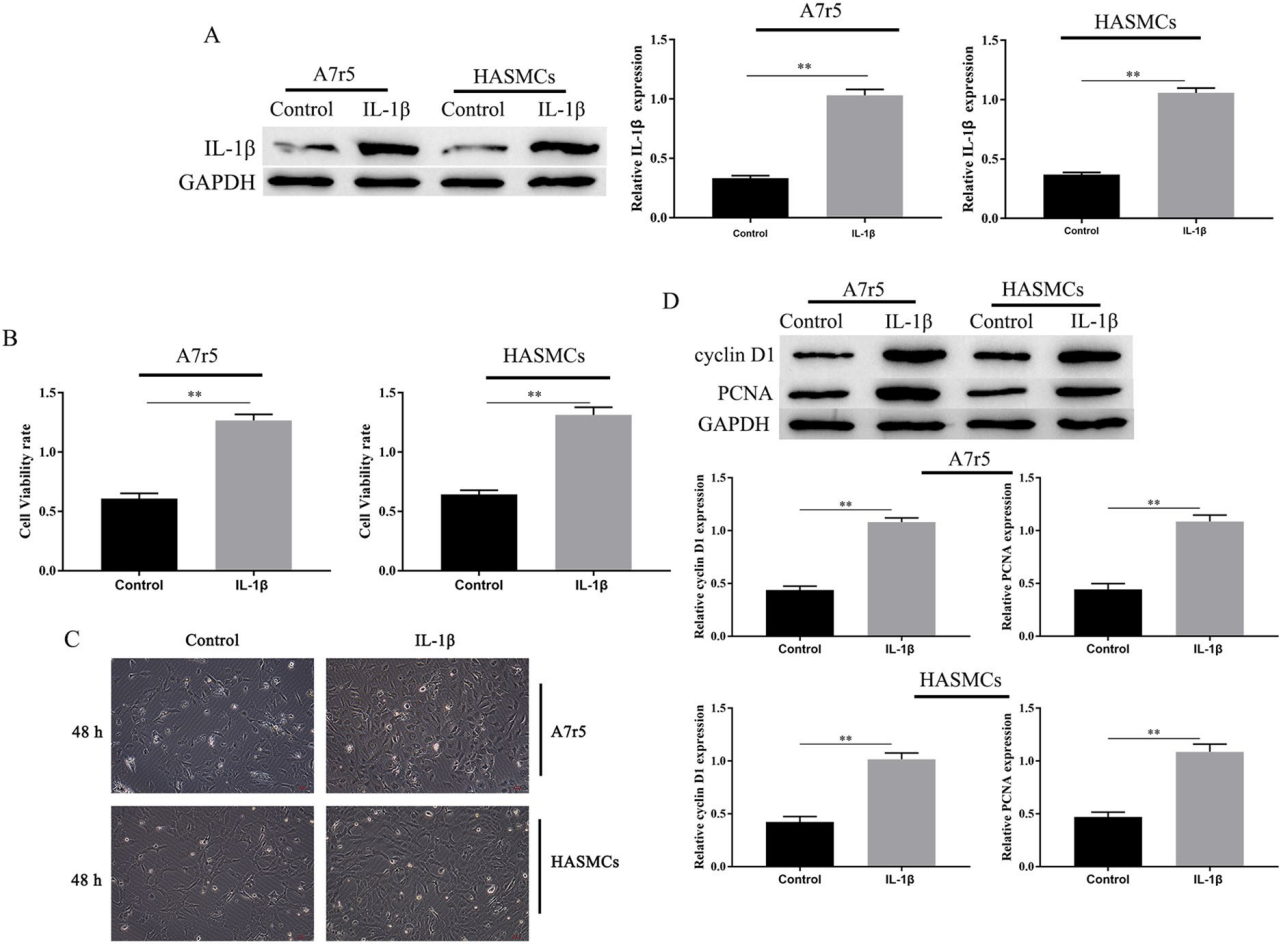
IL-1β treatment promotes the proliferation of A7r5 and HASMCs. The expression of IL-1β, Cyclin D1 and PCNA in A7r5 and HASMCs cells were measured by western blotting (**A**) and (**D**). A7r5 and HASMCs cells viability was measured by the MTT assay and cells viability was observed by microscopic (100 ×) (**B**, **C**).^∗∗^ was considered significant compared to control group (*P* < 0.01(**))

First, A7r5 and HASMCs were treated with IL-1β (10 ng/mL) for 24 h, western blotting results showed that IL-1β expression increased (Fig. 1A). Then, MTT measured cell viability, the results showed that IL-1β significantly promotes the in vitro proliferation of A7r5 and HASMCs (the number of cells inoculated per well was 2 × 10^4^. Figure 1B, C). Additionally, we investigated the expression of proliferation-related proteins PCNA and cyclin D1 by western blotting. Results showed that cyclin D1 and PCNA in A7r5 and HASMCs were significantly upregulated, after IL-1β treatment for 24 h (Fig. 1D). The above results indicate that IL-1β promoted proliferation of A7r5 and HASMCs.

### *IL-1β treatment promotes the invasion and migration of A7r5 and HASMCs *in vitro

**Fig. 2:**
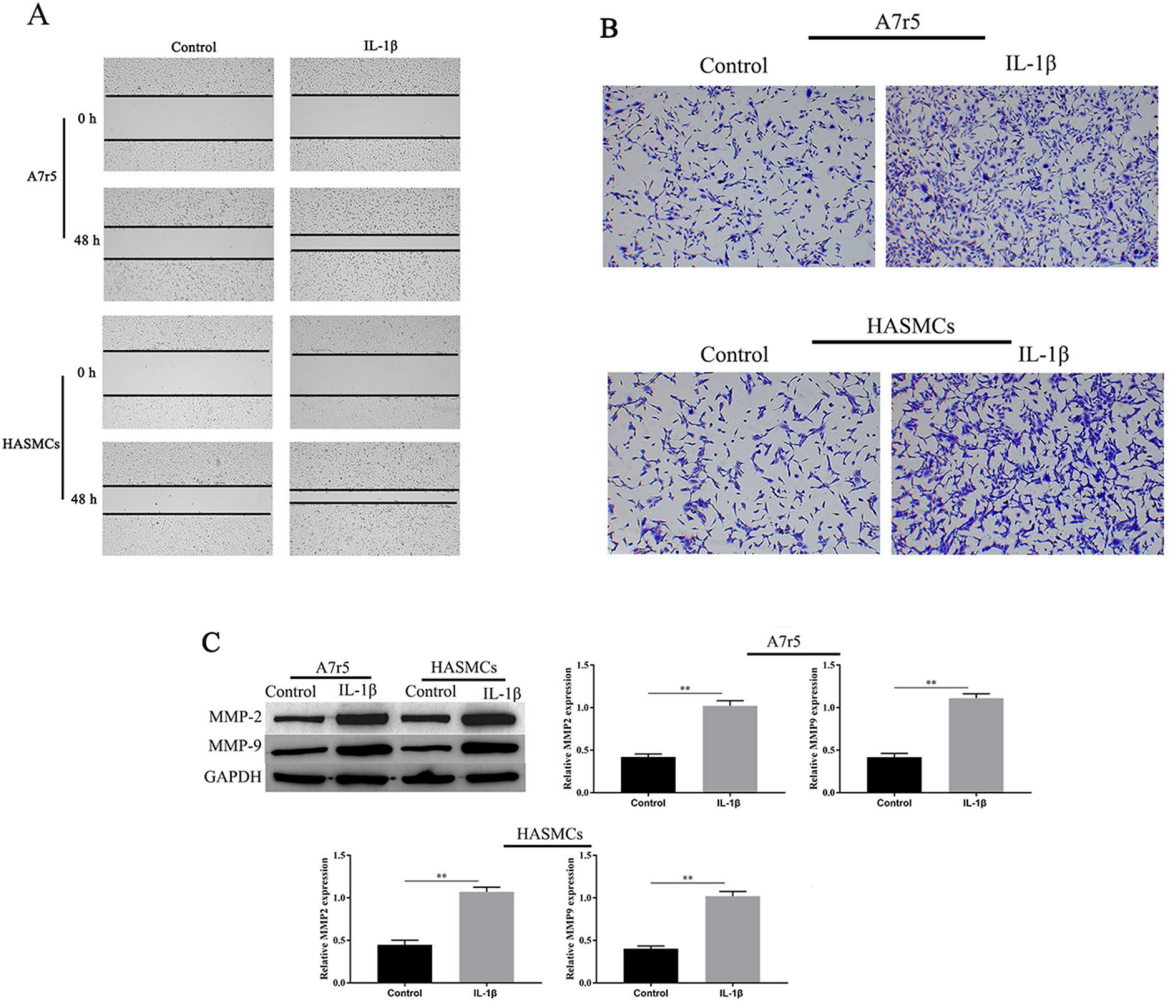
IL-1β treatment promotes the invasion and migration of A7r5 and HASMCs. A7r5 and HASMCs cells migration and invasion ability were measured by Transwell and wound healing assays (× 100) (**A**, **B**). The expression of MMP2, and MMP9 in A7r5 and HASMCs cells was measured by western blotting (**C**). ^∗∗^ was considered significant compared to control group (*P* < 0.01(**))

To further confirm the effect of IL-1β on A7r5 and HASMCs migration and invasion, we investigated cell migration and invasion by wound healing and Transwell assay. The results showed that IL-1β significantly facilitated the A7r5 and HASMCs cells migration and invasion compared with the control group (Fig. 2A, B). Migration-related proteins MMP2 and MMP9 have been confirmed to be involved in the migration and invasion of VSMCs [15]. Therefore, we further detected the effect of IL-1β on the expression levels of MMP2 and MMP9. As shown in Fig. 2C, IL-1β treatment increased MMP2, MMP9 expression levels in A7r5 and HASMCs cells. Thus, IL-1β treatment promoted the invasion and migration, and upregulation of MMP2 and MMP9 are crucial for IL-1β-induced A7r5 and HASMCs cell migration and invasion.

### Angpt-2 promotes IL-1β-induced invasion and migration of A7r5 and HASMCs

**Fig. 3:**
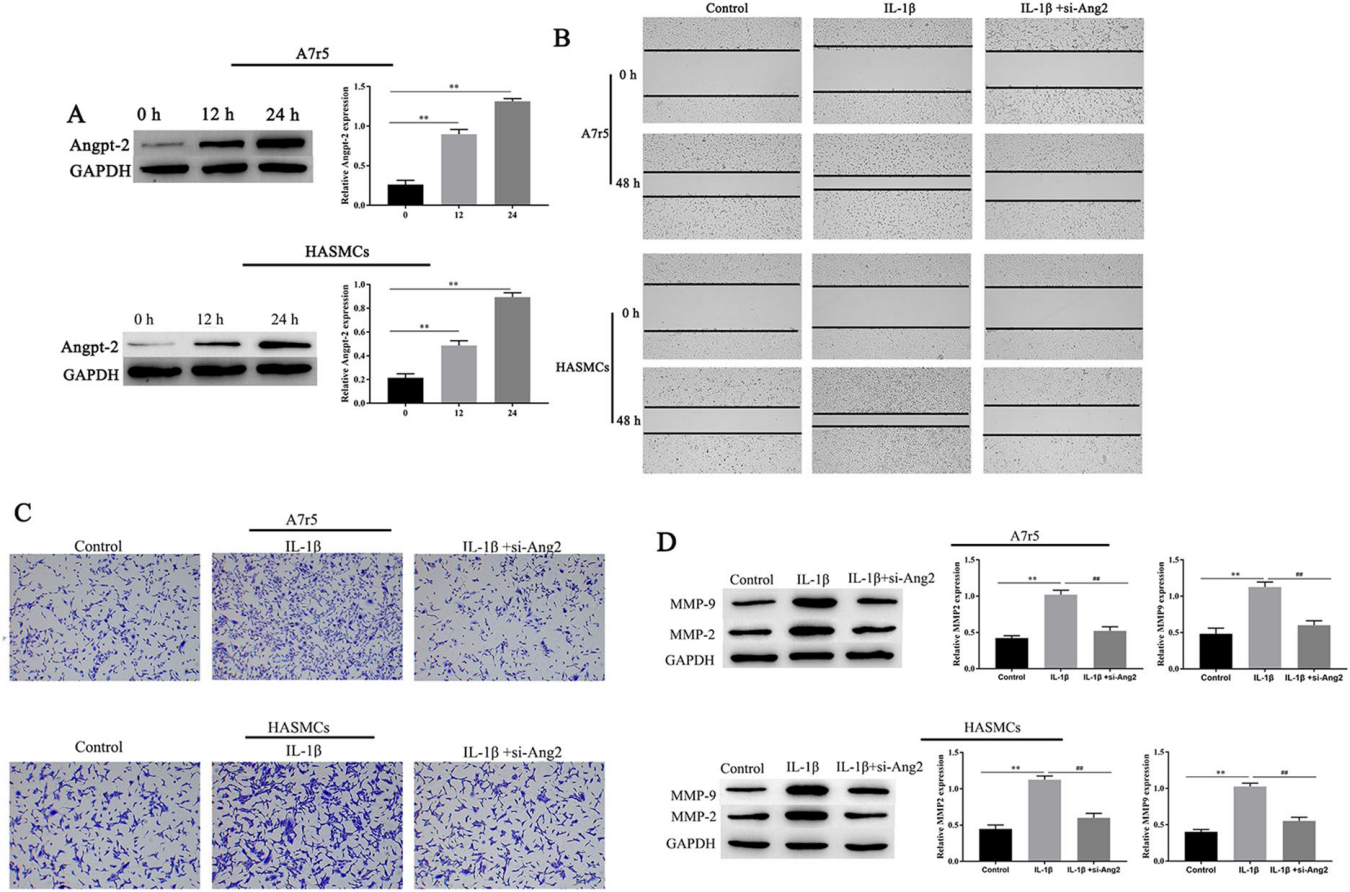
The effects of IL-1β on Angpt-2 expression in A7r5 and HASMCs. The expression of Angpt-2, MMP2, and MMP9 in A7r5 and HASMCs cells was measured by western blotting (**A**) and (**D**). A7r5 and HASMCs cells migration and invasion ability were measured by Transwell and wound healing assays (× 100) (**B**, **C**–c). ^∗∗^ was considered significant compared to control group. ^##^ was considered significant compared to si-Angpt-2 group (*P* < 0.01(**,^##^))

We further studied the molecular mechanism by which IL-1β promotes cell migration and invasion. Next, we investigated whether IL-1β could induce Angpt-2 expression in VSMCs, as in Fig. 3A. In A7r5 and HASMCs treated with IL-1β, the expression of Angpt-2 increased in a time-dependent manner. In addition, si-Angpt-2 significantly inhibited the A7r5 and HASMCs cells migration and invasion compared with the IL-1β group (Fig. 3B, C). Meanwhile, western blotting analysis presented that si-Angpt-2 decreased MMP2, MMP9 expression levels in A7r5 and HASMCs cells (Fig. 3D). The results suggest that Angpt-2 plays a major role in IL-1β-induced migration and invasion.

### Effect of IL-1β on the activation of p38-MAPK in A7r5 and HASMCs

**Fig. 4:**
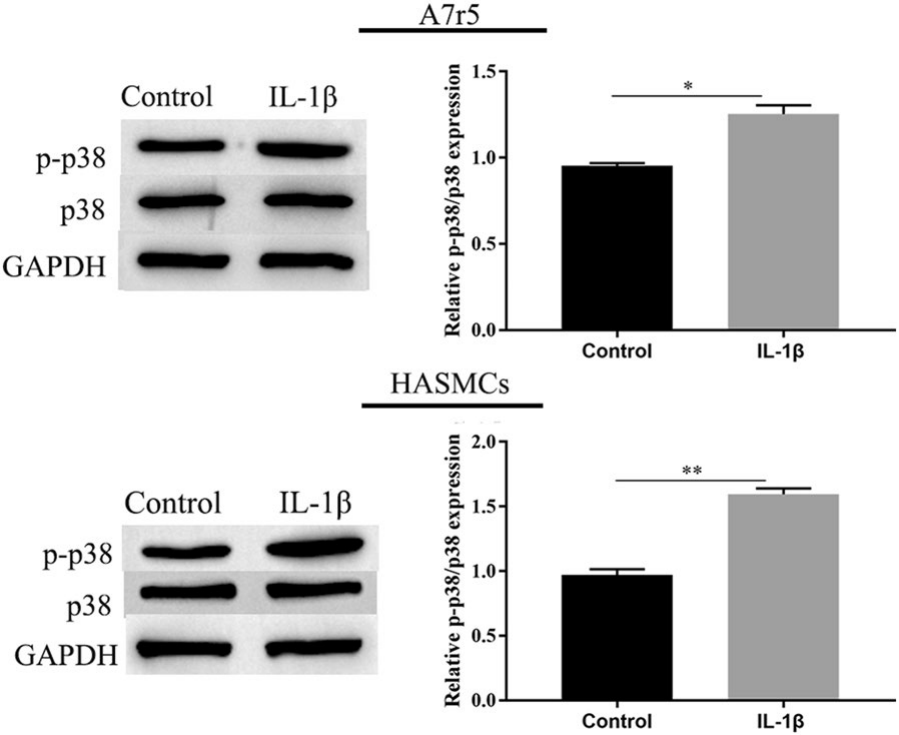
IL-1β activating the p38/MAPK pathway. The expression of p-p38, and p38 in A7r5 and HASMCs was measured by western blotting. ^∗∗^ was considered significant compared to control group (*P* < 0.01(**))

We measured the expression of p38-MAPK pathway. We examined whether IL-1β could induce p38-MAPK pathway. A7r5 and HASMCs were treated with IL-1β (10 ng/mL) for 24 h, western blotting results showed that p-p38 is significantly upregulated in IL-1β-induced A7r5 and HASMCs, as in Fig. 4. Thus, p38-MAPK is activated by IL-1β in A7r5 and HASMC cells.

### IL-1β promoted cell invasion and migration by activating p38-MAPK to upregulate Angpt-2

**Fig. 5:**
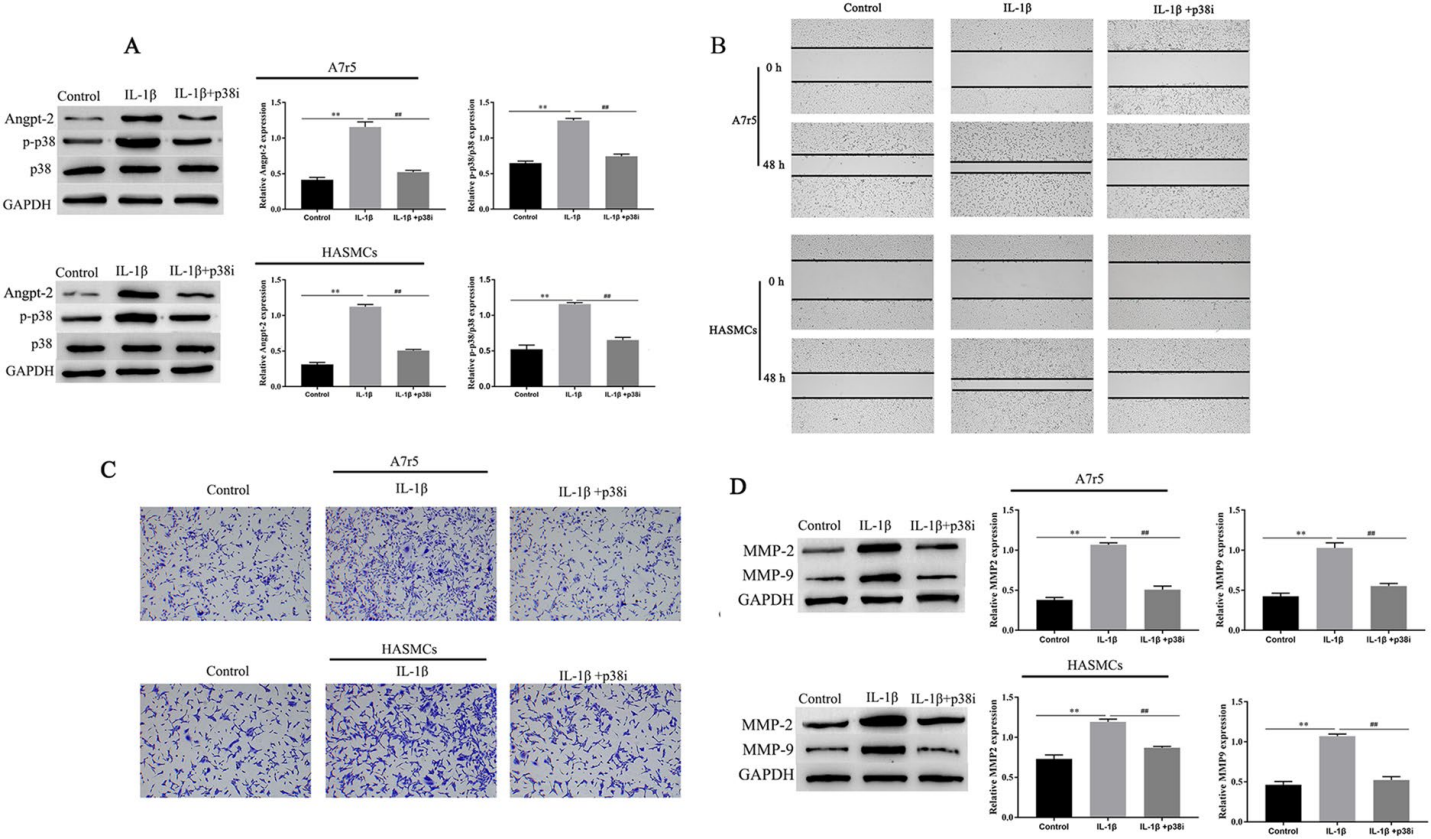
IL-1β promoted cell Invasion and migration by activating p38 MAPK to upregulate Ang2. The expression of Ang2, p-p38, p38, MMP2, and MMP9 in A7r5 and HASMCs cells was measured by western blotting (**A**) and (**D**). A7r5 and HASMCs cells migration and invasion ability were measured by Transwell and wound healing assays (× 100) (**B**, **C**). ^∗∗^ was considered significant compared to control group. ^##^ was considered significant compared to p38i group (*P* < 0.01(**,^##^))

In order to further confirm the roles of p38-MAPK and Angpt-2 in IL-1β-induced Invasion and migration, cells were pretreated with p38-MAPK inhibitor (SB203580 20 nM; p38i) for 6 h. Western blotting showed that p38i treatment significantly reduced the expression of Angpt-2 and p-p38, compared with the IL-1β group (Fig. 5A). Additionally, p38i treatment significantly inhibited the A7r5 and HASMCs cells migration and invasion compared with the IL-1β group (Fig. 5B, C). Simultaneously, western blotting analysis showed that p38i treatment decreased MMP2, MMP9 expression levels in A7r5 and HASMCs cells (Fig. 5D). The above results suggest that IL-1β promoted A7r5 and HASMCs invasion and migration by activating p38-MAPK to upregulate Angpt-2.

## Discussion

VSMCs are the major cell type in the artery. VSMCs migration, proliferation, and inflammatory factor secretion are involved in the important pathological processes of several vascular occlusive diseases, including CAS, and are involved in all stages of CAS [16, 17]. The migration and proliferation of VSMCs are regulated by cytokines, growth factors, and other stimuli. IL-1β, as a bioactive mediator of VSMC synthesis and secretion, can promote the pathological progress of CAS [11]. In this study, we further explored the mechanism by which IL-1β regulates VSMC proliferation, migration and obtained several findings. First, IL-1β treatment promotes the proliferation, invasion and migration of A7r5 and HASMCs, and upregulated the expression of cyclin D1, PCNA, MMP2 and MMP9. Second, our results indicate that Angpt-2 is upregulated after IL-1β treatment and mediates the effect of IL-1β on the invasion and migration of A7r5 and HASMCs. Third, we demonstrated for the first time that the IL-1β promotes A7r5 and HASMCs invasion and migration by activating p38-MAPK to upregulate Angpt-2.

Matrix metalloproteinase (MMP), a class of zinc-dependent proteinases, has important function in tissue remodeling, wound healing and cardiovascular diseases through regulated degradation of the ECM and facilitating extracellular matrix [18, 19]. Degradation of ECM is required for cell migration [20]. In CAS, MMP promotes VSMC proliferation and migration by inducing ECM degradation and remodeling [21]. Among them, MMP2 and MMP9 are the key to mediate the degradation of ECM and regulate the migration of VSMC [22]. In the present study, we found that after IL-1β treatment, in addition to increased invasion and migration of VMSCs, the expression of MMP2 and MMP9 was also upregulated. This suggests that IL-1β promotes the activation and secretion of MMPs, especially MMP2 and MMP9. The results were consistent with Eun et al. [11].

Angpt-2, as a peptide that promotes VSMC proliferation, migration, oxidative stress, inflammation and vascular remodeling, plays a key role in regulating the function of VSMC [23]. In VSMC-mediated degradation and remodeling of ECM, Angpt-2 inhibitor disrupted the integrity of VSMCs and inhibited the function of VSMCs [24]. In addition, plasma levels of Angpt-2 reflect different pathophysiological aspects of vascular occlusive and cardiovascular disease [25]. It is reported that elevated Angpt-2 has been observed in patients with vascular occlusive diseases including CAS [26, 27]. Angpt-2 is expressed and released from HUVEC, and mediates endothelial inflammation to initiate atherosclerosis and angiogenesis [27]. Simultaneously, exogenous Ang2 promotes HUVEC migration, adhesion and tube formation with similar potency to VEGF [28]. On the other hand, the level of Angpt-2 is also correlated with IL-1β [29]. Thus, we wondered whether IL-1β regulates VSMC proliferation and migration by regulating Angpt-2. In this study, we found that in A7r5 and HASMCs treated with IL-1β, the expression of Angpt-2 increased in a time-dependent manner. Simultaneously, Angpt-2 is involved in IL-1β-induced A7r5 and HASMCs proliferation and migration. In addition, IL-1β-regulated MMP2 and MMP9 secretion is also closely associated with the expression of Angpt-2. Thus, our results suggest that Angpt-2 plays a major role in IL-1β-induced migration and invasion, and promotes the secretion and expression of MMP2 and MMP9. However, the mechanism by which IL-1β regulates Angpt-2 is still unclear.

A previous study found that p38-MAPK inhibitor blocks insulin-induced Angpt-2 expression and Angpt-2 secretion [27]. This gave us a hint that p38-MAPK may be involved in the regulation of cell migration and invasion by Angpt-2. MAPK pathway, as a key signal pathway affecting cell migration and invasion, plays a role by responding to extracellular stimuli [30]. In the process of inflammation, the p38-MAPK signaling pathway has been confirmed to be activated by IL-1β to promote inflammation [12]. In addition, p38-MAPK play a crucial role in regulating the biosynthesis of IL-1β and TNF-α [31]. In this study, we confirmed that p38-MAPK is activated by IL-1β in A7r5 and HASMC cells. This finding is consistent with a previous study [12]. Parallelly, we found that p38-MAPK inhibitors can affect the upregulation of Angpt-2 induced by IL-1β, and further participate in the secretion of MMP2 and MMP9, and affect A7r5 and HASMCs invasion and migration.

**Fig. 6:**
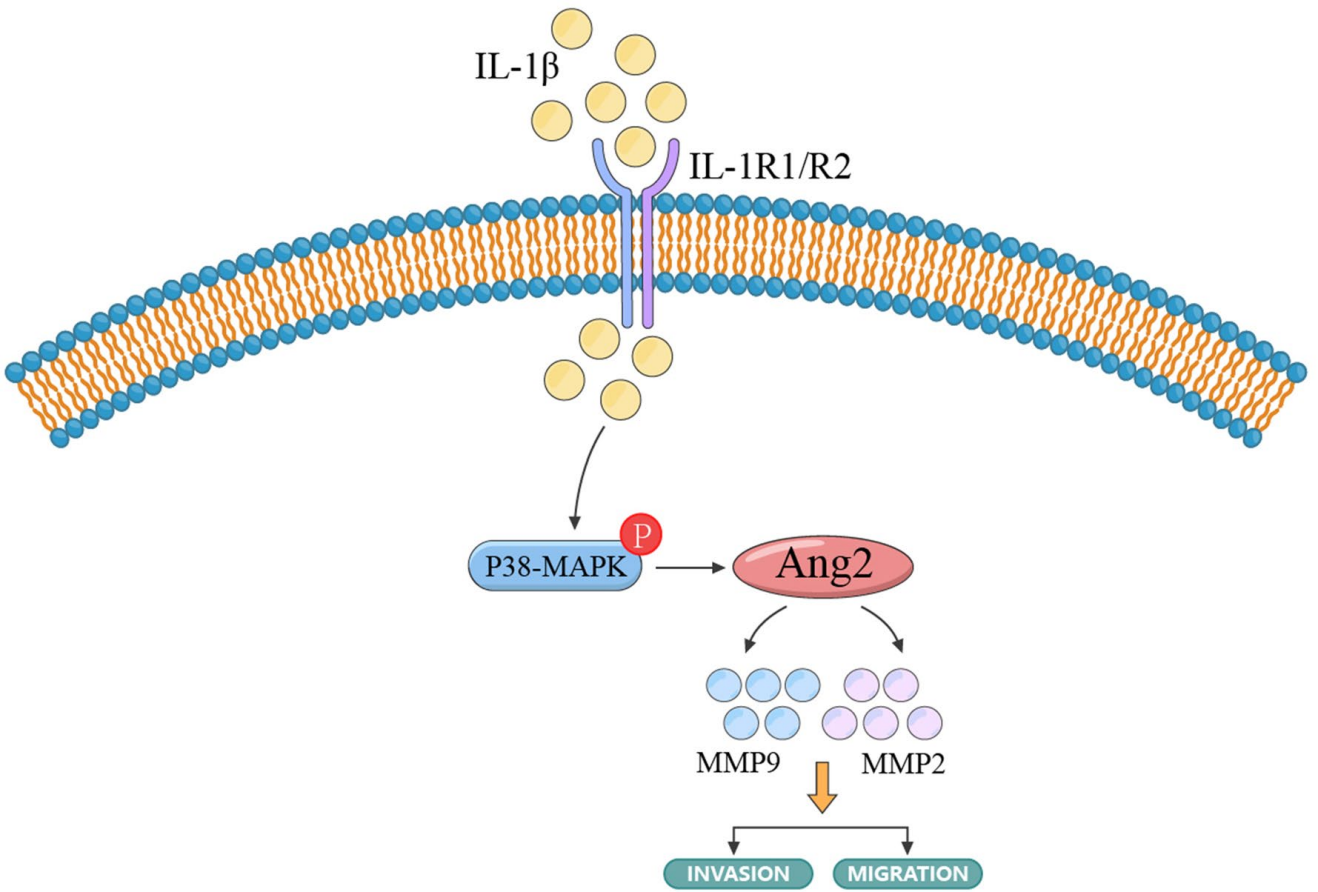
Schematic diagram of IL-1β regulating the invasion and migration of A7r5 and HASMCs. IL-1β activates the p38-MAPK pathway in cells, and the activation of p38-MAPK promotes the expression of Angpt-2, which further activates MMP2 and MMP9 and promotes their secretion. The release of MMP2, MMP9 promotes the invasion and migration of A7r5 and HASMC

In conclusion, our results indicate that IL-1β induces the expression of Angpt-2 in A7r5 and HASMC, and secretes MMP2 and MMP9 to promote cell invasion and migration. Subsequently, the p38-MAPK pathway is activated in cells induced by IL-1β. Inhibition of the p38-MAPK pathway can downregulate the expression of Angpt-2 and inhibit cell invasion and migration (Fig. 6).

## Data Availability

The data used to support the findings of this study are available from the corresponding author upon reasonable request.
